# Toward a Differential Diagnosis of Hidden Hearing Loss in Humans

**DOI:** 10.1371/journal.pone.0162726

**Published:** 2016-09-12

**Authors:** M. Charles Liberman, Michael J. Epstein, Sandra S. Cleveland, Haobing Wang, Stéphane F. Maison

**Affiliations:** 1 Department of Otology and Laryngology, Harvard Medical School, Boston, MA, United States of America; 2 Eaton-Peabody Laboratory, Massachusetts Eye & Ear Infirmary, Boston, MA, United States of America; 3 Harvard Program in Speech and Hearing Bioscience and Technology, Boston, MA, United States of America; 4 Department of Communication Sciences and Disorders, Bouvé College of Health Sciences, Northeastern University, Boston, MA, United States of America; Universidad de Salamanca, SPAIN

## Abstract

Recent work suggests that hair cells are not the most vulnerable elements in the inner ear; rather, it is the synapses between hair cells and cochlear nerve terminals that degenerate first in the aging or noise-exposed ear. This primary neural degeneration does not affect hearing thresholds, but likely contributes to problems understanding speech in difficult listening environments, and may be important in the generation of tinnitus and/or hyperacusis. To look for signs of cochlear synaptopathy in humans, we recruited college students and divided them into low-risk and high-risk groups based on self-report of noise exposure and use of hearing protection. Cochlear function was assessed by otoacoustic emissions and click-evoked electrocochleography; hearing was assessed by behavioral audiometry and word recognition with or without noise or time compression and reverberation. Both groups had normal thresholds at standard audiometric frequencies, however, the high-risk group showed significant threshold elevation at high frequencies (10–16 kHz), consistent with early stages of noise damage. Electrocochleography showed a significant difference in the ratio between the waveform peaks generated by hair cells (Summating Potential; SP) vs. cochlear neurons (Action Potential; AP), i.e. the SP/AP ratio, consistent with selective neural loss. The high-risk group also showed significantly poorer performance on word recognition in noise or with time compression and reverberation, and reported heightened reactions to sound consistent with hyperacusis. These results suggest that the SP/AP ratio may be useful in the diagnosis of “hidden hearing loss” and that, as suggested by animal models, the noise-induced loss of cochlear nerve synapses leads to deficits in hearing abilities in difficult listening situations, despite the presence of normal thresholds at standard audiometric frequencies.

## Introduction

Most hearing impairment in adults is sensorineural in origin. It is caused by damage to the inner ear, where the cochlear hair cells normally convert mechanical vibrations into electrical signals that are transmitted via glutamatergic synapses to the sensory fibers of the cochlear nerve. Each human cochlea contains only ~15,000 hair cells and ~40,000 nerve fibers. Once destroyed, neither cell type regenerates in any mammalian ear [1].

Decades of research on noise-exposed humans and animals have shown that acoustic overexposure leads to hair cell damage, which in turn causes threshold elevation (e.g. [2, 3]). The dogma has been that hair cells are the primary targets of noise and that cochlear neurons only die as a result of hair cell degeneration [4]. This view arose because hair cell loss can be detected within hours post noise exposure, while loss of spiral ganglion cells is not detectable for months to years after the insult [5, 6]. According to this view, a noise exposure that only causes a temporary elevation of cochlear thresholds is benign, because there is no permanent hearing impairment. This assumption underlies the damage-risk criteria for noise in the workplace set by several federal agencies [7].

Recent animal studies showing that noise exposure can lead to cochlear neuronal degeneration, even when hair cells recover and thresholds return to normal [8] have challenged this view. In noise-exposed ears showing no acute or chronic hair cell loss, there can be up to a 50% loss of the synapses between inner hair cells (IHCs) and cochlear neurons. The same primary loss of cochlear synapses occurs in the aging ear [9, 10]. This cochlear synaptopathy has remained “hidden” because, although loss of synapses is immediate, the synapses are not visible in routine histological material, and the subsequent loss of spiral ganglion cells takes months to years [11]. Cochlear synaptopathy is also “hidden” because cochlear neural degeneration does not elevate behavioral or electrophysiological thresholds until it becomes extreme [12, 13].

Part of the reason for the relative insensitivity of threshold measures to cochlear synaptopathy is that, near threshold, a small increase in sound level can compensate for a large loss of neurons, by increasing discharge rates in remaining fibers and by spreading activity to additional fibers along the cochlear spiral [14]. Another part of the explanation is that the most vulnerable cochlear neurons, to both noise and aging, are those with high thresholds and low spontaneous rates (SRs) [15, 16]. These low-SR fibers do not contribute to threshold detection in quiet, but, by virtue of their high thresholds, are key to the coding of transient stimuli in the presence of continuous background noise [17] that saturates the responses of the sensitive high-SR fibers. These observations have suggested that low-SR fiber loss is a major contributor to the classic impairment in sensorineural hearing loss (SNHL), i.e. difficulties with speech discrimination in challenging listening environments [18].

Cochlear synaptopathy may also be key to the genesis of other perceptual anomalies associated with noise damage, including hyperacusis and tinnitus [19–23], which may arise via an induction of central gain adjustment secondary to loss of afferent input to the auditory central nervous system [24].

Based on the animal work, we hypothesized that cochlear synaptopathy is widespread among young people who regularly abuse their ears, despite the presence of a normal audiogram. For the present study, we recruited young adult subjects and divided them according to noise-exposure history into high-risk and low-risk groups. We found significant deficits in difficult word-recognition tasks in the high-risk group that were associated with significant elevation of pure-tone thresholds at frequencies higher than those normally tested and with changes in auditory evoked potentials consistent with the presence of cochlear synaptopathy, also known as hidden hearing loss.

## Materials and Methods

### Participants, Groups and Questionnaires

Young adults were recruited from local colleges and universities. Subjects were between 18 and 41 yrs of age, in good health, with no history of ear or hearing problems, and no history of neurologic disorders. They were all native speakers of English with unremarkable otoscopic examinations. To be eligible, participants had to have normal audiometric thresholds from 0.25–8 kHz in both ears. There were no other inclusion criteria beyond the ability to give voluntary informed written consent prior to participation. This research study was reviewed and approved by the Institutional Review Board of Northeastern University.

All subjects completed a series of questionnaires (see S1 Appendix) that included 1) general questions to assess medical history related to ear and hearing and 2) specific questions designed to provide an overall metric of noise-exposure types and durations and whether they *systematically* used hearing protection devices when exposed to loud sounds. Based on their responses, each subject was assigned to a group, low-risk or high-risk for ear damage. Most high-risk subjects were studying music performance in local music colleges and conservatories. Most low-risk subjects were students in communication science and disorders.

To these generic medico-legal, noise and ear health questions, were added 5 items relating to the subject’s self assessment of their hearing abilities in different listening environments (1 item for hearing and listening in quiet and 4 items for listening in “noisy environments” such as those with reverberation, multiple talkers or background noise) and 3 items relating to their ability to localize a sound source. Subjects were asked to answer the question “can you follow the conversation?” by rating their ability on a scale from 0 (not at all) to 10 (perfectly). Participants were then asked to rate separately the loudness and annoyance of 12 everyday sounds (e.g., barking dog next to you, baby crying in the same room, someone sniffing or clearing their throat) on a scale from 0 (not loud at all / not annoying at all) to 100 (unbearably loud / extremely annoying). Finally, participants were asked to rate on a scale from 0 (totally disagree) to 100 (completely agree) with 10 statements related to hyperacusis (e.g., “because sounds are too loud, you avoid shopping”; “because sounds are too loud, you are not able to concentrate”).

All the following tests were administered in a sound-treated booth at the Northeastern University Speech-Language and Hearing Center.

### Audiometric Thresholds and Speech Recognition

Audiometric thresholds were obtained using an Otometrics Madsen^®^ Astera^2^ audiometer. Pure-tone air-conduction thresholds were measured from 250 Hz to 16,000 Hz at octave intervals and also at 9, 10, 11.2, 12.5 and 14 kHz. At 8 kHz and below TDH-39P headphones were used; above 8 kHz, we used a circumaural HDA200 high-frequency headset. Bone-conduction thresholds were acquired from 250 Hz to 4,000 Hz by placing a Radioear B-71 vibrator over the mastoid process of the temporal bone. Word recognition performance was assessed monaurally at 35 dB HL using the Northwestern University Auditory Test Number 6 (NU-6) list consisting of 50 phonemically balanced words. Word recognition scores were assessed in 5 conditions using different word lists for each condition: in quiet or in the presence of ipsilateral noise at a signal-to-noise ratio of 5 dB or 0 dB (3 conditions) or after digital time compression to 45% or 65% of original duration, both with a 0.3 sec reverberation added (2 additional conditions) [25, 26]. In order to maintain the subjects’ attention during speech testing, a 2-min break was given before the presentation of each new list.

### Distortion Product Otoacoustic Emissions

Stimulus generation and data acquisition were handled by a GSI Audera^™^ Distortion Product Otoacoustic Emission (DPOAE) system and software (Grason-Stadler, Eden Prairie, MN). DPOAEs were measured as amplitude vs. frequency sweeps—or DPgrams—with two primary tones f_1_ and f_2_ (f_2_/f_1_ = 1.22), at f_1_ = 65 dB SPL and f_2_ = 55 dB SPL. Primary frequencies were swept from f_2_ = 500 Hz to 12 kHz, with 4 logarithmically spaced steps per octave. The DPOAE at 2f_1_-f_2_ was extracted from the ear canal sound pressure after both waveform and spectral averaging, without corrections for standing wave artifacts.

### Electrocochleography

Stimulus generation and data acquisition were handled by a GSI Audera^™^ AEP/CAEP computer system and software (Grason-Stadler, Eden Prairie, MN). Subjects’ ear canals were prepped by scrubbing with a cotton swab coated in Nuprep^®^. Electrode gel was applied on the clean portion of the canal and over the gold-foil of each tiptrode before insertion. A horizontal montage was used with a ground on the forehead at midline, one tiptrode as the inverting electrode and the other tiptrode as the non-inverting electrode in the opposite ear. Impedance between pairs of electrodes were <2 kΩ. Acoustic stimuli were delivered via silicone tubing connected to the ER-3A earphones. Stimuli were 100 μs-clicks delivered at 94.5 dB nHL in alternating polarity at a rate of 9.1 Hz or 40.1 Hz. Ipsilateral clicks were presented with a 55 dB nHL contralateral broadband masker to eliminate contamination of the ABR responses with contributions from the contralateral ear. Electrical responses were amplified 100,000X with a 10–3,000 Hz passband filter. Up to 2,000 sweeps were averaged, with artifact rejection enabled in the software.

The summating potential (SP) and action potential (AP) peaks were defined by visual inspection by two observers (one blinded to experimental groups): SP placements were identical in 98% of cases; AP placements were identical in all cases. The SP peak was defined as the highest inflection point within the first msec post-stimulus onset, i.e. where the slope either approached or reached zero. The AP peak was placed at the maximum value between 1 and 2 msec post onset: in all but two cases (both from the high-risk group) the AP peak was between 1 and 1.5 msec. SP and AP amplitudes were defined as the difference between peak and baseline (lowest amplitude within the first msec).

### Statistics

Two-tailed heteroscedastic t-tests were used to test the statistical significance of the mean differences observed between our two groups. Two-way ANOVAs were used to compare the mean differences between groups when there were two independent variables. To test for the significance of the correlation coefficient, we computed the t value as follow: t = r √ ((n-2)/(1-r^2^)) with degrees of freedom equal to n-2.

## Results

### Audiometry, otoacoustic emissions and self report

We performed behavioral threshold audiometry, electrophysiology and gave word recognition tests to 34 young adult participants, divided into two groups: low-risk or high-risk for ear damage based on self-report of exposure to loud sound and the systematic use of hearing protection. Most high-risk subjects (n = 22, age: 25 yrs ±1.3, 7 females– 15 males) were studying music performance in local colleges and conservatories. Most low-risk subjects (n = 12, age: 24 yrs ± 0.9, 8 females– 4 males) were studying communication sciences and disorders and were knowledgable about the damaging effects of noise/music overexposure on the inner ear. None of these subjects was familiar with the content of the NU-6 word list.

**Fig 1:**
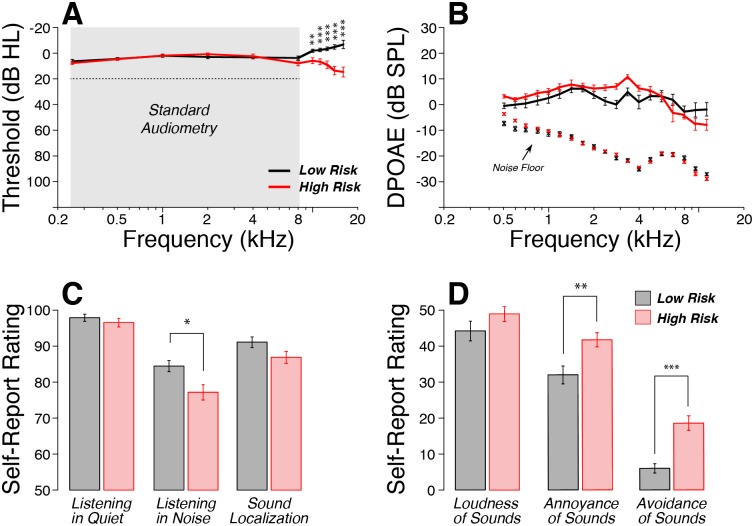
A: All subjects had normal thresholds (< 20 dB HL) at standard audiometric frequencies (≤ 8 kHz), however the high-risk group showed significant threshold elevation at all high frequencies tested (10–16 kHz). B: Mean DPOAE amplitudes were not significantly different between the groups: L_1_ and L_2_ were 65 and 55 dB SPL, respectively. C: According to self-report, there were no significant intergroup differences in perceived ability to hear in quiet or to localize sounds, however the high-risk group, on average, reported more difficulty hearing in noisy backgrounds. D: In describing their reactions to 12 everyday sounds, the high-risk group rated them as louder, and more annoying, and scored higher on the questions designed to reveal hyperacusis. All data are means (±SEM). * p<0.05, **p<0.01, ***p<0.001.

All subjects had normal audiometric thresholds (< 20 dB HL) across the standard audiometric frequencies (0.25–8 kHz), and mean hearing thresholds were closely matched between groups (Fig 1A). However, the high-risk group showed significant threshold elevation at all test-frequencies above 8 kHz, growing to ~20 dB HL at 16 kHz: the intergroup differences were statistically significant for each of these high-frequency pure tones (0.01<p<0.001).

Outer hair cell (OHC) function was evaluated by recording distortion product otoacoustic emissions (DPOAEs), which are created and amplified in the healthy inner ear, and propagated back to the ear canal, where they can be measured in the sound pressure recorded in response to two continuous tones in the appropriate frequency and level ratios [27]. DPOAEs were present in all subjects and across test-frequencies, and their amplitudes were not significantly different between groups (Fig 1B, p>0.05).

According to the questionnaires, no participants felt they had hearing difficulties in a quiet environment. All subjects rated their performance as close to “perfect” with average scores > 95% in both groups (Fig 1C). Although both groups reported poorer performances in noisy backgrounds, ratings were significantly lower in the high-risk group (77%) than the low-risk group (85%) (p<0.05). Similarly, the high-risk group reported slightly more difficulties localizing sound sources in challenging environments, but differences with the low-risk group were not statistically significant. When comparing self-report values directly to mean high-frequency thresholds, without parsing subjects into groups, the only significant correlation was that between poorer thresholds and more reported difficulty with sound localization (S1 Fig).

When asked to rate the loudness and annoyance of 12 everyday sounds, subjects in the high-risk group tended to give higher values. However, only differences in annoyance ratings reached statistical significance (Fig 1D; p<0.01). High-risk subjects were more likely to report behavior consistent with hyperacusis, i.e. the tendency to avoid noisy environments of different types (Fig 1E, p<0.001). Self-report of hyperacusis was not significantly correlated with mean high-frequency thresholds (S1 Fig). There were no significant inter-group differences in the numbers of subjects reporting having experienced tinnitus at least on one occasion.

### Electrocochleography

**Fig 2:**
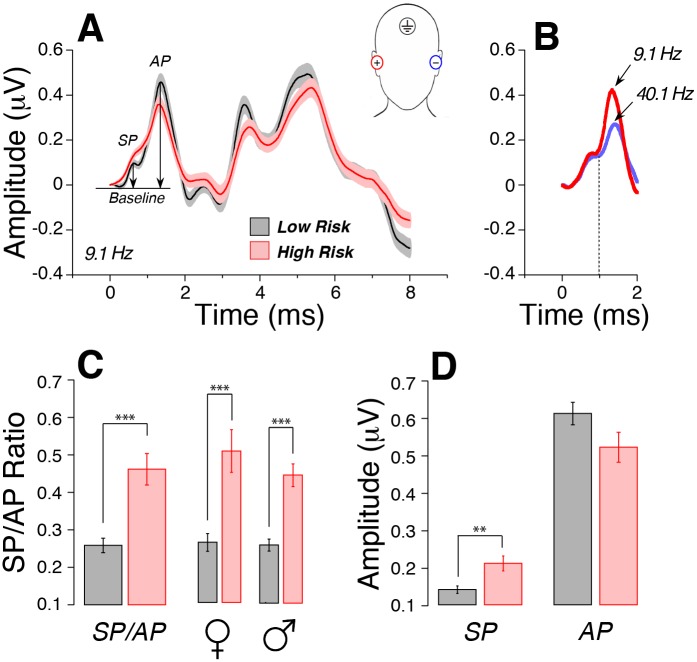
Electrocochleography shows evidence for cochlear synaptopathy in the high-risk group. A Averaged waveforms (±SEMs) from each group in response to clicks delivered at 9.1 Hz in alternating polarity at 94.5 dB nHL. SP and AP are measured from baseline to peak, as illustrated. B: Increasing click rate from 9.1 Hz to 40.1 Hz decreases AP without affecting SP: mean waveforms from 6 subjects are shown. C: Mean SP/AP ratio is nearly twice as high in the high-risk vs. the low-risk group. This difference remains when subjects are separated by sex. D: The difference in SP/AP ratios arises from both an increase in the SP and a decrease in the mean AP, although only the SP differences are statistically significant. All data are means (±SEM). ***p<0.001; **p<0.01.

To assess cochlear neural function, we measured click-evoked auditory brainstem responses from “tiptrode” electrodes in the ear canal (Fig 2). Animal work has shown that the suprathreshold amplitude of wave 1 of the auditory brainstem response (ABR), which represents the summed activity of the cochlear nerve fibers, can be diagnostic of cochlear synaptopathy [8]. Because wave 1 in humans, as measured via conventional ABR electrode configurations, is small and variable, we used ear-canal electrodes, where wave 1, a.k.a. the AP (action potential) is larger [28], and where a pre-synaptic summating potential (SP), can also be measured [29]. In animal work on both noise-induced and age-related synaptopathy, SP amplitudes remain robust, while the AP amplitude is reduced by accumulating neural damage [9]. We reasoned that, by taking the ratio between SP and AP in our human subjects, we could eliminate some of the variability in human electrophysiology that arises from inter-subject differences in head size, electrode contact, etc.

Fig 2A shows the mean electrocochleographic traces from each group in response to alternating-polarity clicks at 94.5 dB nHL. Averaged waveforms from both groups had absolute and inter-peak latencies within normal limits. Early responses (< 2 ms) obtained from all subjects showed clearly defined SP and AP waves. Increasing the click rate from 9.1 Hz to 40.1 Hz (Fig 2B) did not alter the SP amplitude, while it significantly decreased the AP amplitude. This is consistent with the idea that the SP is dominated by hair cell receptor potentials, which do not adapt to this type of click train, whereas the AP is generated by the cochlear nerve, which does adapt as click rate is increased [30].

Individual SP/AP ratios were computed and averaged across groups. The high-risk group had a mean SP/AP ratio nearly twice that of the low-risk group (Fig 2C, 0.46 vs. 0.26), and the intergroup differences were highly significant (p<0.001). Given the known effect of gender on auditory evoked response amplitudes [31, 32], SP/AP ratios were averaged separately for each sex: intergroup differences remained highly significant despite the relatively small size of each group (Fig 2C, p<0.001). Surprisingly, the difference in SP/AP ratios reflected both a decrease in AP amplitude and an increase in SP amplitude (Fig 2A and 2D), and only the difference in SP amplitude was statistically significant (Fig 2D, p<0.01).

### Word recognition

**Fig 3:**
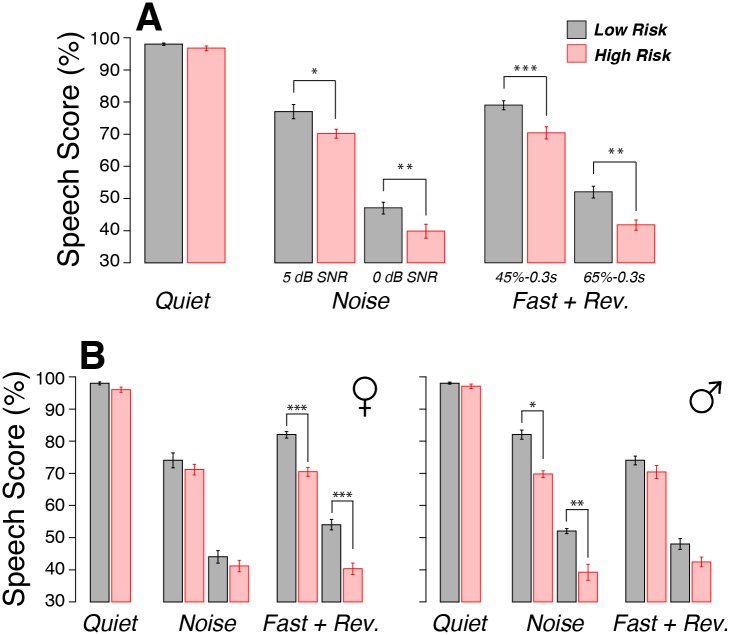
Word recognition performance was significantly poorer in the high-risk group in the presence of noise, or time compression plus reverberation. A: Mean word-recognition scores for 50 NU-6 words presented monaurally at 35 dB HL either (left) in *Quiet*, or (middle) with ipsilateral white noise at signal-to-noise ratios of 5 dB or 0 dB (*Noise*), or (right) with time compression of 45% or 65% and a reverberation time of 0.3 sec (*Fast + Rev*.). B: Same data as A, but separated by sex as indicated. All data are means (±SEM). * p<0.05, **p<0.01, ***p<0.001.

Of the many speech-in-noise tests used in clinical studies [33], we chose the NU-6 corpus, because these lists of phonemically balanced words offer no contextual clues. When a NU-6 word list was presented monaurally at 35 dB HL in quiet, speech recognition scores were excellent (>96%) and similar between groups (Fig 3A). When NU-6 word lists were presented in the presence of ipsilateral white noise, the high-risk group performed more poorly at both a signal-to-noise ratio of 5 dB (Fig 3A, p<0.05) and 0 dB (Fig 3A, p<0.01). Similarly, when NU-6 words were time compressed (45% or 65%) and a reverberation time of 0.3 sec was added, intergroup differences were larger and statistically significant for both compression ratios (Fig 3A, 0.01<p<0.001). Some significant differences remained when groups were further separated by sex (Fig 3B).

**Fig 4:**
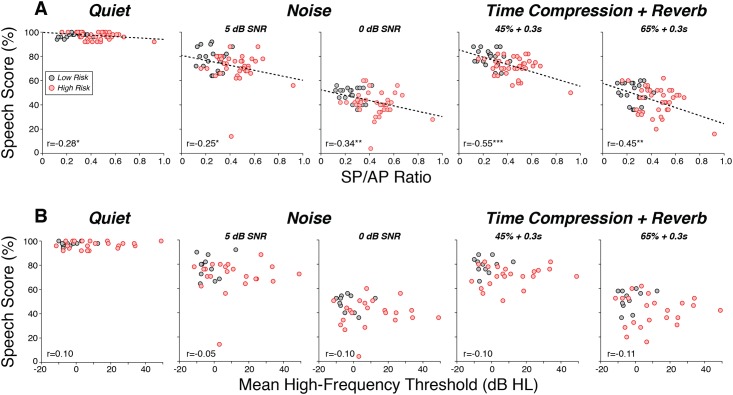
Speech scores were better correlated with the SP/AP ratio (A) than with the mean high-frequency threshold elevation (B). Data are the same as those from Fig 3, except that values for each subject are shown. Dashed lines show regression lines for those correlations that were statistically significant. * p<0.05, **p<0.01, ***p<0.001.

To gain insight into whether the performance decrements were due to synaptopathy or the high-frequency threshold shifts, we compared the correlations between individual speech scores and the SP/AP ratio (Fig 4A) to the correlations seen between speech scores and mean high-frequency thresholds (9–16 kHz; Fig 4B). The comparison suggests that it is not the high-frequency threshold shift that is the cause of the problems hearing in difficult listening situations: high-frequency thresholds were uncorrelated with word-recognition performance for any of the listening conditions, whereas the SP/AP ratios were significantly correlated with performance for all four of the non-quiet conditions. Removing the “outlier” subject (with an SP/AP ratio > 0.9) only eliminates significance for the 5 dB signal-to-noise condition: the significance remained for the 0 dB signal-to-noise case (p = 0.027) and for both time compression conditions (p = 0.002 for 45% and p = 0.027 for 65%).

## Discussion

### The importance of high-frequency audiometry

Recent animal studies of the noise-exposed or aging ears have shown that synaptic connections between hair cells and cochlear neurons can degenerate well before the loss of sensory cells themselves. This cochlear synaptopathy has been called “hidden hearing loss”, in part, because it can hide behind a normal audiogram [23]. Animal experiments have shown that diffuse cochlear neural degeneration does not elevate behavioral thresholds, as long as hair cell function is normal [13, 34]. Although cochlear synaptopathy has little effect on the thresholds for pure tones in a quiet environment, it likely compromises the ability to understand complex stimuli, like speech, in difficult listening situations [35].

This study aimed to test the hypothesis that “hidden hearing loss” is widespread among young adults with normal audiometric thresholds, especially those who abuse their ears regularly. We identified and recruited college students and divided them into high-risk and low-risk groups based on self-report of exposure to loud sounds and use of hearing protection devices. Both subject groups had normal audiometric thresholds up to 8 kHz, as this was one of the entry criteria. Standard audiometric testing does not assess thresholds above 8 kHz, although younger people can easily hear tone frequencies at least an octave higher (16 kHz). We chose to measure high-frequency thresholds (from 8–16 kHz), because animal studies show that, regardless of the frequency spectrum of the traumatic sound, the first signs of noise-induced hair cell damage and threshold elevation are at the extreme cochlear base, i.e. in cochlear regions tuned to the very highest frequencies for that species [11, 36, 37]. Only as the damage worsens, do threshold become elevated at frequencies closer to those in the exposure stimulus.

Indeed, our high-risk subjects showed significant threshold elevations at all test frequencies > 8 kHz, reaching ~20 dB HL on average at 16 kHz (Fig 1A). This high-frequency threshold elevation is consistent with early noise damage, and thus suggests that the self-report data obtained from participants captured important aspects of their noise exposure history. It also suggests that high-frequency audiometry can provide early warning of ear abuse, which might inspire regular use of hearing protection and the prevention of further noise-induced damage and the mounting sensory impairment that it will produce. This is especially important given evidence from animal studies that early noise exposure causes an acceleration of subsequent age-related cochlear degeneration [38, 39].

### Cochlear synaptopathy and the SP/AP ratio

In animals, cochlear synaptopathy can be diagnosed via the suprathreshold amplitudes of ABR wave 1, when the interpretation is not complicated by hair cell damage and the threshold shift that it causes [9, 39]. In human studies, inter-subject variability in ABR amplitude, due to the small signal-to-noise ratio and the heterogeneity in head shape/size, tissue conductivity, electrode resistance etc., likely complicates its diagnostic utility [40]. We hoped to reduce this variability by 1) recording from a site closer to the generators (i.e. using tiptrodes inserted deep into the ear canal) and 2) by normalizing ABR wave 1 (or AP) to the hair-cell generated summating potential (or SP). In prior mouse work on cochlear synaptopathy caused by genetic manipulations [41], noise-induced damage [8], age-related changes [9] or drug-induced neuropathy [42], the stability of SP amplitude in the face of decreasing AP has been noted. Similarly, humans with auditory neuropathy due to mutations in otoferlin, a protein essential for synaptic transmission between hair cells and cochlear neurons, show a robust SP and a minimal AP [43].

Indeed, although intergroup differences in AP amplitudes were not significant in the present study (Fig 2C), the SP/AP ratio was significantly affected in our high-risk group (Fig 2D). Surprisingly, there was also a significant enhancement of the SP, *per se*, in the high-risk group, which clearly contributed to the intergroup differences in SP/AP ratio. These results are strikingly similar to those in a prior study comparing SP and AP amplitudes before and immediately after exposure to music traumatic enough to cause a temporary threshold shift of 10–15 dB [44]. In that study, AP amplitudes declined while SP amplitudes increased. In both studies, the electric responses represent the summation of equal numbers of stimuli of opposite polarity in order to remove the large “microphonic” potentials generated by the hair cell receptor currents.

Interpretation of the electric response in and around the SP peak is complicated, because it could have contributions from two types of potentials: 1) non-linear components in the receptor potentials from inner and/or outer hair cells which are not cancelled by stimulus reversal, and 2) excitatory post-synaptic potentials (EPSPs) from cochlear nerve terminals under inner hair cells (which don’t change polarity with stimulus reversal). Both these contributions can have negative or positive components. Thus, for example, enhancement of the SP could arise from loss of a negative EPSP rather than from the enhancement of a positive hair cell non-linearity. It is also possible that the putative synaptopathy in the high-risk group has attenuated the middle-ear muscle reflex, as observed in synaptopathic mice [45]. This reflex attenuation would increase the effective click level in the high-risk group, enhancing both the SP and the AP, thereby minimizing the AP reduction, but leaving the SP/AP ratio a more robust measure of synaptopathy.

Although an increased SP/AP ratio in the high-risk group is consistent with cochlear synaptopathy, possible contribution of the basal-turn hair cell damage inferred from the high-frequency threshold elevations (Fig 1A) must also be considered. It is unlikely that these high-frequency cochlear regions were being stimulated even in the low-risk group, because the ER-3A earphones used to deliver the click stimuli have little energy above ~4 kHz, and the overall SPL is likely not high enough to stimulate the low-frequency “tails” of high-frequency cochlear neurons [46, 47]. Furthermore, if the click were stimulating the basal-turn hair cells, the damage should decrease both SP and AP. The most compelling reason to doubt a contribution of high-frequency hair cell damage to the perceptual and electrophysiological abnormalities in the high-risk group is the lack of significant correlation between individual high-frequency thresholds and speech recognition scores (Fig 4). Nonetheless, the most convincing way to eliminate this confound in future studies will be to add a high-pass masking noise to remove any contributions from high-frequency cochlear neurons to the electrophysiological responses.

Prior human work has suggested that the SP/AP ratio might be diagnostic for Ménière’s disease [28], an inner ear disorder characterized by fluctuating threshold shifts, vertigo and tinnitus, and thought to be due to increased fluid pressure in the endolymphatic spaces. In the past, speculations on the mechanisms underlying the correlation between enhanced SP/AP and Ménière’s focused on possible changes in mechano-electric transduction due to static pressure changes across the sensory epithelium. However, an enhanced SP/AP ratio in Ménière’s may actually be due to synaptopathy: electron microscopic studies showed dramatic de-afferentation of IHCs in a case of unilateral Ménière’s disease: there were only ~3 synapses per IHC in the affected ear vs. ~12 synapses per IHC in the “normal” ear of this post-mortem specimen [48].

### Perceptual correlates of cochlear synaptopathy

It is well know that two people with the same audiogram, whether normal or abnormal, can have different speech discrimination abilities, especially in a noisy environment [33, 49], and the contribution of cochlear neurodegeneration to this difference in impairment has always been a logical possibility. However, recent animal work has led to the novel idea that significant de-afferentation of surviving IHCs may be the rule rather than the exception in acquired sensorineural hearing loss and that significant hair cell de-afferentation occurs well before elevation of audiometric thresholds in the noise-exposed and/or aging ear [18].

Here, we show that our high-risk subjects report increased difficulty in noisy environments (Fig 1C) and have poorer speech discrimination scores when the task is made difficult by adding masking noise, or time-compression and reverberation (Fig 3). This type of difficulty understanding speech in difficult listening environments is a very common complaint among the hearing impaired. Prior animal work has suggested that such performance decrements might arise because noise-induced cochlear synaptopathy preferentially destroys the subset of cochlear nerve fibers with high thresholds and low-SRs [16] and because, by virtue of their high thresholds, low-SR fibers can continue to respond to tone bursts in the presence of continuous noise that completely masks the tone-evoked response of low-threshold high-SR fibers [17]. However, others have suggested that, since the response patterns of cochlear neurons are probabilistic in nature, subtotal neuronal loss will cause problems hearing in noise due to a kind of stochastic undersampling of the stimulus waveform [35], regardless of which subtype of neurons is destroyed.

Recent human studies suggest beneficial effects of musical training on speech-in-noise abilities [50, 51]. Thus, considering that 70% of participants from our high-risk group were active musicians, it is possible that our study underestimates intergroup differences in underlying cochlear synaptopathy. The reported central effects of intense musical training on speech-in-noise abilities would tend to minimize the detrimental effects of peripheral pathology.

Sensorineural hearing loss, especially in aging ears, is often accompanied by tinnitus and hyperacusis. It has been suggested that loss of sensory outflow from the auditory periphery causes a compensatory increase in “central gain” that underlies these perceptual anomalies [24, 52]. Indeed, animal work has suggested that cochlear synaptopathy, in the absence of hair cell loss, may lead to both tinnitus and hyperacusis [19, 20, 23]. Although here we saw no increase in self-report of tinnitus among our high-risk group, there was a significant increase in the degree of sound annoyance and avoidance among those subjects with higher SP/AP ratios.

### Early detection and future treatments

Present data suggest that a combination of ear-canal electrocochleography, high-frequency audiometry and word recognition tasks can possibly identify the earliest signs of noise damage to hair cells and neurons, neither of which is detected by standard audiometry. In animal studies of noise-induced damage, mid-cochlear synaptopathy is seen at exposure levels lower than those that damage the basalmost hair cells and elevate high-frequency thresholds [8]. Thus, in noise-damaged humans, we expect that high-frequency threshold elevation will be correlated with mid-cochlear synaptopathy. Indeed, in the present study, the correlation between SP/AP ratio and mean high-frequency thresholds was highly significant (p<0.001; S2 Fig).

Noise damage early in life likely accelerates the age-related further loss of hair cells and cochlear neurons, even in the absence of further ear abuse [8, 39]. Clarification of the true risks of noise, and the true prevalence of noise-induced damage, are important to public policy on noise abatement, to raising general consciousness about the dangers of ear abuse and to preventing a dramatic rise in hearing impairment in the future.

Although the noise-induced loss of cochlear synaptic connections can happen within hours post-exposure [53], the degeneration of the cell bodies and central projections of these cochlear neurons takes years to decades [54]. Thus, there may be a long therapeutic window within which the sensory cells and sensory neurons could be reconnected. Indeed, recent animal research has reported regeneration of cochlear nerve synaptic connections with inner hair cells after noise exposure, along with corresponding functional recovery, using neurotrophin-based therapies, either via genetically driven overexpression [55] or via local delivery of neurotrophic factors directly to the round window membrane of the inner ear [56]. Future clinical trials of such regenerative therapies will require objective measures of cochlear synaptopathy to identify candidates and to track treatment efficacy.

## Supporting Information

S1 Appendix Questionnaires. (DOC)

S1 Fig Self-report performance scores as a function of SP/AP ratio or mean High-Frequency Threshold. (TIF)

S2 Fig Mean High-Frequency Threshold as a function of SP/AP ratio. (TIF)
