# Supplementary material for: Toward a Differential Diagnosis of Hidden Hearing Loss in Humans
Source: PLoS One. 2016 Sep 12;11(9):e0162726. doi: 10.1371/journal.pone.0162726 (PMC5019483; doi:10.1371/journal.pone.0162726)
Supplement: S1 Appendix — (DOC) [file pone.0162726.s001.doc]

**Appendix 1**

***Questionnaire 1***

(1) Have you ever had tubes put into your eardrum(s)? (circle one)

yes no

(2) Have you ever had a ruptured ear drum? (circle one)

yes no

If yes, at what age ________________________________

Which ear was affected (circle one): left right both

(3) Have you ever had a job where you were routinely exposed to very loud sounds, such as military experience with artillery, construction or factory work? (circle one)

yes no

If yes, at what age(s) and for how long? ________________________________

About what percentage of the time did you use ear protection? _____________

Please describe the job(s). __________________________________________

(4) Have you had any hobbies that involved loud sounds, such as hunting, riding motorcycles, carpentry, frequently attending concerts? (circle one)

yes no

If yes, at what age(s) and for how long? ________________________________

About what percentage of the time did you use ear protection? _____________

Please describe the hobby(s). _________________________________________

(5) Have you ever experienced temporary noises (ringing, buzzing, hiss, swishing, etc…) in your ears after exposure to loud sounds? (circle one)

yes no

If yes, how often do you experience these noises (occasionally, constantly)

________________________________

(6) Have you ever temporarily lost your hearing? (circle one)

yes no

If yes, at what age(s) and for how long? ________________________________

What was the cause? _______________________________________________

(7) Do sounds ever seem distorted to you? (circle one)

yes no

If yes, please explain. _______________________________________________

(8) When you are having a conversation in a noisy place, such as a restaurant or bar, can you understand what is being said as well as everyone else can? (circle one)

yes no

If no, please explain. _______________________________________________

(9) Have you had sinus problems or sinus surgery? (circle one)

yes no

If yes, please explain. _______________________________________________

(10) Have you had a major blow to the head or any other head trauma? (circle one)

yes no

If yes, please answer the following. If you’ve had more than one instance of head trauma, please

answer for each time it occurred.

How old were you? _________________

What happened? _________________

Were you diagnosed with a concussion? _________________

**Circle any of the following which accompanied the head trauma:**

Loss of consciousness (even briefly). How long: _________________

Bleeding from the ears

Tinnitus

Memory or concentration problems

Headaches or weakness persisting days afterward

Dizziness or balance problems persisting days afterward

(11) Have you had a whiplash injury, as might happen in a car collision? (circle one)

yes no

If yes, please explain. _______________________________________________

(12) Have you ever been diagnosed with temporomandibular joint disorder (TMJ)? (circle one)

yes no

(13) Do places on or near your ear hurt when you press on them? (circle one)

yes no

If yes, on which side? (circle one) right left both

(14) Have you ever experienced a sharp pain in the ear or an aching ear that was not from an ear infection? (circle one)

yes no

If yes, on which side? (circle one) right left both

If yes, when and for how long? _____________________________________________

Did any event seem to trigger these symptoms? ________________________________

(15) Have you ever experienced a sensation of “ear fullness” or “blockage” and or muffled hearing that lasted for more than a few minutes and was not due to a pressure change (such during a plane flight, for instance)? (circle one)

yes no

If yes, on which side? (circle one) right left both

If yes, when and for how long? _____________________________________________

Did any event seem to trigger these symptoms? ________________________________

(16) Have you ever experienced numbness, a burning sensation, or pain along the side of your neck, around your ear, or in your cheek? (circle one)

yes no

If yes, on which side? (circle one) right left both

If yes, when and for how long? _____________________________________________

Did any event seem to trigger these symptoms? ________________________________

(17) Have you been told you grind your teeth when you sleep? (circle one)

yes no

(18) Have you ever been diagnosed with any of the following? (circle all that apply)

a. Generalized Anxiety Disorder (GAD)

b. Panic Disorder

c. Obsessive-Compulsive Disorder (OCD)

d. Posttraumatic Stress Disorder (PTSD)

e. Bipolar Disorder

f. Major Depressive Disorder

g. Schizophrenia

h. Schizoaffective Disorder

i. Brief Psychotic Disorder

j. Dissociative Amnesia, or Fugue

k. Dissociative Identity Disorder

l. Depersonalization Disorder

m. Intermittent Explosive Disorder

n. Kleptomania

o. Anorexia

p. Bulimia

q. Body Dysmorphic Disorder

r. Conversion Disorder

s. Hypochondriasis Disorder

t. Pain Disorder

u. Somatization Disorder

v. Adjustment Disorder

w. Substance abuse and/or dependence

x. Alzheimer’s

y. Parkinson’s

z. Huntington’s

aa. Meniere’s

bb. Fibromyalgia

cc. Irritable Bowel Syndrome

dd. Multiple Sclerosis

ee. Migraine

ff. Autism spectrum

gg. Attention Deficit or Attention Deficit Hyperactivity Disorders (ADD/ADHD)

hh. Other neurological or psychiatric disorder (please list)

(19) How often do you experience balance problems? (circle one)

never sometimes often always

If often or always, please explain. _____________________________________

(20) Do certain sounds irritate you more than they would other people?

yes no

If yes, (a) list the sounds and (b) rate how irritating each sound is on a 0 (not at all) to 100 (extremely) scale:

_______________________________________________________________________

(21) Are you bothered by bright lights? (circle one)

yes no

If yes, rate how much on a 0 (not at all) to 100 (extremely bothersome) scale: ______

(22) Are you bothered by certain colors? (circle one)

yes no

If yes, rate how much on a 0 (not at all) to 100 (extremely bothersome) scale: ______

(23) Are you bothered by strong smells? (circle one)

yes no

If yes, rate how much on a 0 (not at all) to 100 (extremely bothersome) scale: ______

(24) Are you bothered by certain smells? (circle one)

yes no

If yes, rate how much on a 0 (not at all) to 100 (extremely bothersome) scale: ______

(25) Are you bothered by certain tastes? (circle one)

yes no

If yes, rate how much on a 0 (not at all) to 100 (extremely bothersome) scale: _____

(26) Are you bothered by certain types of touch? (circle one)

yes no

If yes, rate how much on a 0 (not at all) to 100 (extremely bothersome) scale: ______

(27) How many years of education have you completed (beyond kindergarten)? (circle one)

1. less than 6 years
2. 6-8 years
3. 9-12 years
4. 13-14 years
5. 15-16 years
6. more than 16 years

(28) What is the nature of your current job? (Circle all that apply)

1. Accounting / Finance / Banking
2. Athletics
3. Artist / Author
4. Software Developer/Computer Programmer
5. IT / Web Developer
6. Sound engineer
7. Engineer (other than sound)
8. Law Enforcement/ Firefighting
9. Construction
10. Pilot / Truck Driver
11. Human Resources/ Management
12. Lawyer
13. Current or Former Military
14. Musician
15. Medicine/ Pharmacy/ Oral Health
16. Psychologist/Social worker
17. Public Relations/Advertising
18. TV/ Radio/ Journalist
19. Researcher (please specify area e.g., humanities, physics etc.)
20. Teacher/Lecturer
21. Service Industry/ Retail
22. Student
23. Homemaker
24. Between jobs
25. Retired
26. Other. Please specify: _______________________

(29) Is your hearing particularly important for your job or for activities you do often?

Or for any other research studies you may have participated in recently?

(some examples: birding, sound engineer, other research studies on hearing, playing a musical instrument).

Yes No

If yes, please describe the activities and the role hearing plays (for example, “I have to listen for faint sounds.”):

(30) Approximately how often do you listen for very faint sounds, listen for slight differences between sounds, or concentrate on the pitch or other features of sounds?

a. Never

b. Almost never

c. Occasionally

d. More than occasionally, but less than 1 hour per day

e. Between 1 and 4 hours per day

f. Between 4 and 8 hours per day

g. More than 8 hours per day

(31) Are you currently under any medication? If so, please specify:_________________________

***Questionnaire 2***

The following questions ask about your ability and experience hearing and listening in different situations.

For each question, put a mark, such as a cross (X), anywhere on the scale that runs from 0

through 10, below each question. Putting a mark at 10 means you would be perfectly able to do or experience what is described in the question. Putting a mark at 0 means you would be unable to do or experience what is described.

***We expect that all the questions are relevant to your everyday experience, but if a question describes a situation that does not apply to you, put a cross in the “not applicable” box.***

1) You are in a group of about five people, sitting round a table. It is an otherwise quiet place. You can see everyone else in the group. Can you follow the conversation?

***Not at all*** 0---1---2---3---4---5---6---7---8---9---10 ***Perfectly* ☐*Not Applicable***

2) You are talking with one other person. There is continuous background noise, such as a fan or running water close by. Can you follow what the person says?

***Not at all*** 0---1---2---3---4---5---6---7---8---9---10 ***Perfectly* ☐*Not Applicable***

3) You are in a group of about five people in a busy restaurant. You CANNOT see everyone else in the group. Can you follow the conversation?

***Not at all*** 0---1---2---3---4---5---6---7---8---9---10 ***Perfectly* ☐*Not Applicable***

4) You are talking to someone in a place where there are a lot of echoes, such as a church or railway station. Can you follow what the other person says?

***Not at all*** 0---1---2---3---4---5---6---7---8---9---10 ***Perfectly* ☐*Not Applicable***

5) You are having a conversation with one person in a room where there are many other people talking. Can you follow what the person you are talking to is saying?

***Not at all*** 0---1---2---3---4---5---6---7---8---9---10 ***Perfectly* ☐*Not Applicable***

6) You are sitting around a table or at a meeting with several people. You can’t see everyone. Can you tell where any person is as soon as they start speaking?

***Not at all*** 0---1---2---3---4---5---6---7---8---9---10 ***Perfectly* ☐*Not Applicable***

7) You are sitting in between two people. One of them starts to speak. Can you tell right away whether it is the person on your left or your right, without having to look?

***Not at all*** 0---1---2---3---4---5---6---7---8---9---10 ***Perfectly* ☐*Not Applicable***

8) You are in an unfamiliar house. It is quiet. You hear a door slam. Can you tell right away where that sound came from?

***Not at all*** 0---1---2---3---4---5---6---7---8---9---10 ***Perfectly* ☐*Not Applicable***

***Questionnaire 3***

***Part 1***

*Some everyday sounds are loud and some are soft. Some everyday sounds are annoying*

*and some are not. Please rate the* ***loudness*** *and the* ***annoyance*** *of the following sounds.*

*Do not consider the annoyance when rating the loudness and do not consider the*

*loudness when rating the annoyance. For example, a sound may be very loud, but not*

*annoy you. Likewise, a sound may be very soft, yet be very annoying. Rate the sounds*

*using a scale from 0 (not loud/annoying) to 100 (unbearable loud/annoying).*

**Sound Loudness Annoyance**

(0 – 100) (0 – 100)

| 1. Barking dog next to you. |  |  |
| --- | --- | --- |
| 2. Dishes clanking together in the same room |  |  |
| 3. Music on the radio in a car when the volume is adjusted for normal-hearing listeners |  |  |
| 4. Music on the radio in a quiet room when the volume is  adjusted for normal-hearing listeners |  |  |
| 5. Telephone ringing in the same room |  |  |
| 6. Television in the same room when the volume is  adjusted for normal-hearing listeners |  |  |
| 7. Lawn mower running next to you |  |  |
| 8. Car door closing next to you |  |  |
| 9. Someone talking to you in a noisy restaurant |  |  |
| 10. Baby crying in the same room |  |  |
| 11. Someone chewing food or gum |  |  |
| 12. Someone sniffing or clearing their throat |  |  |

***Part 2***

*The following questions relate to hearing loss, tinnitus and hyperacusis. Hyperacusis is*

*when sounds that are moderately loud for other people are* ***too*** *loud for you.*

*Please rate your agreement/disagreement with the following statements, using a scale*

*from 0 (completely disagree) to 100 (completely agree):*

**Because sounds are too loud (0-100)**

| 1. You avoid shopping |  |
| --- | --- |
| 2. You do not go out with your friends |  |
| 3. You have given up some hobbies |  |
| 4. You do not go to restaurants |  |
| 5. You avoid being in crowds |  |
| 6. You feel depressed |  |
| 7. You feel anxious |  |
| 8. You are not able to concentrate |  |
| 9. Your quality of life is poor |  |
| 10. You are not able to perform tasks  or jobs as well |  |
