# Supplementary figures and images for: Toward a Differential Diagnosis of Hidden Hearing Loss in Humans
Source: PLoS One. 2016 Sep 12;11(9):e0162726. doi: 10.1371/journal.pone.0162726 (PMC5019483; doi:10.1371/journal.pone.0162726)

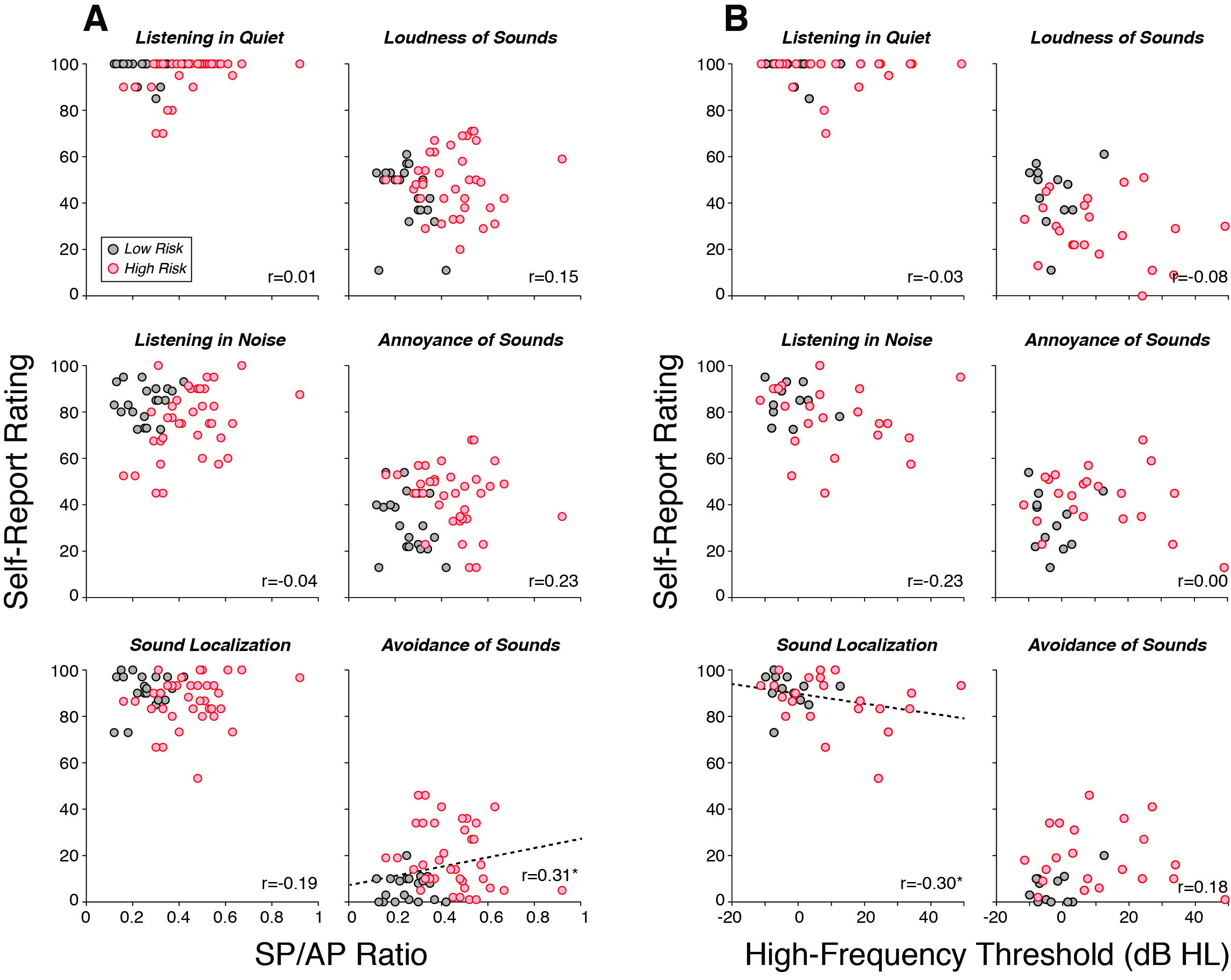

Supplement: S1 Fig — (TIF) [file pone.0162726.s002.tif]

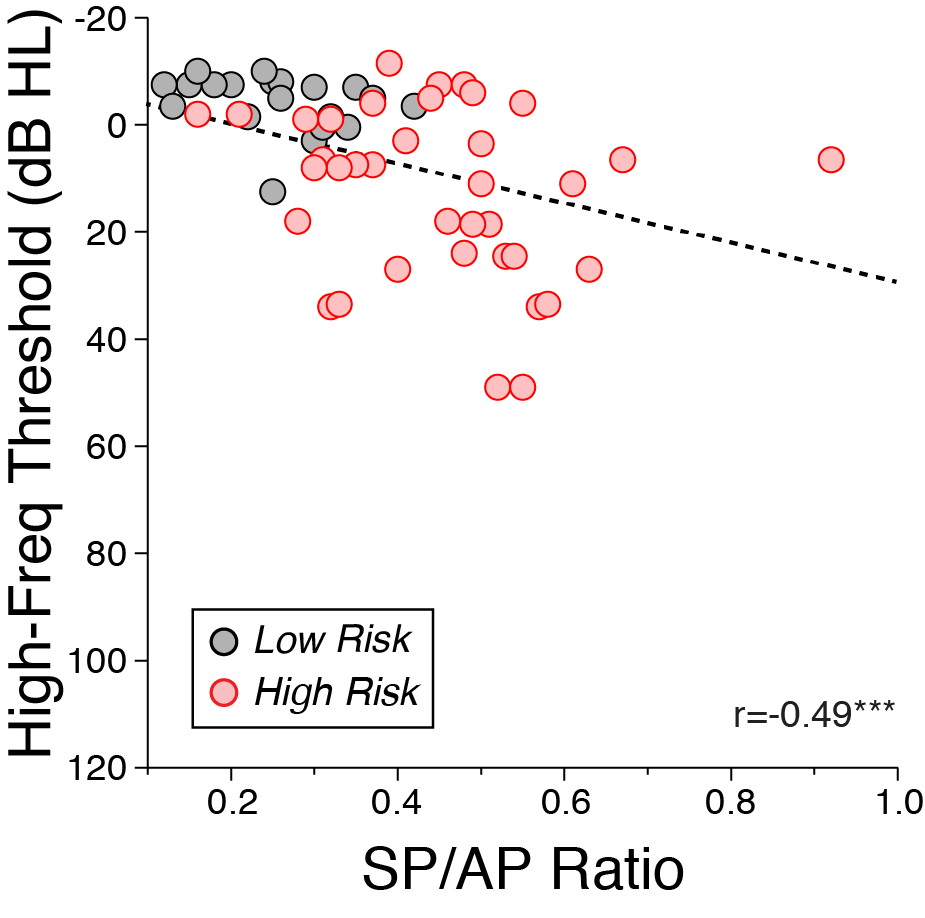

Supplement: S2 Fig — (TIF) [file pone.0162726.s003.tif]
